# A Non-Invasive Approach for Facial Action Unit Extraction and Its Application in Pain Detection

**DOI:** 10.3390/bioengineering12020195

**Published:** 2025-02-17

**Authors:** Mondher Bouazizi, Kevin Feghoul, Shengze Wang, Yue Yin, Tomoaki Ohtsuki

**Affiliations:** 1 Faculty of Science and Technology, Keio University, Yokohama 223-8522, Japan; yin@ohtsuki.ics.keio.ac.jp (Y.Y.); ohtsuki@ics.keio.ac.jp (T.O.); 2 Graduate School of Science and Technology, Keio University, Yokohama 223-8522, Japan; feghoul@ohtsuki.ics.keio.ac.jp (K.F.); wang@ohtsuki.ics.keio.ac.jp (S.W.)

**Keywords:** 3D facial landmarks, action units, pain detection, transformer

## Abstract

A significant challenge that hinders advancements in medical research is the sensitive and confidential nature of patient data in available datasets. In particular, sharing patients’ facial images poses considerable privacy risks, especially with the rise of generative artificial intelligence (AI), which could misuse such data if accessed by unauthorized parties. However, facial expressions are a valuable source of information for doctors and researchers, which creates a need for methods to derive them without compromising patient privacy or safety by exposing identifiable facial images. To address this, we present a quick, computationally efficient method for detecting action units (AUs) and their intensities—key indicators of health and emotion—using only 3D facial landmarks. Our proposed framework extracts 3D face landmarks from video recordings and employs a lightweight neural network (NN) to identify AUs and estimate AU intensities based on these landmarks. Our proposed method reaches a 79.25% F1-score in AU detection for the main AUs, and 0.66 in AU intensity estimation Root Mean Square Error (RMSE). This performance shows that it is possible for researchers to share 3D landmarks, which are far less intrusive, instead of facial images while maintaining high accuracy in AU detection. Moreover, to showcase the usefulness of our AU detection model, using the detected AUs and estimated intensities, we trained state-of-the-art Deep Learning (DL) models to detect pain. Our method reaches 91.16% accuracy in pain detection, which is not far behind the 93.14% accuracy obtained when employing a convolutional neural network (CNN) with residual blocks trained on actual images and the 92.11% accuracy obtained when employing all the ground-truth AUs.

## 1. Introduction

Facial expressions serve as a fundamental component in enabling emotion-aware human communication and deepening our understanding of emotions. By providing essential cues, they play an important role in helping us interpret the thoughts, intentions, and emotions of others. In this context, facial action units (AUs) were introduced in the Facial Action Coding System (FACS) [1] to quantify facial expressions, allowing researchers to build robust and reproducible rules for emotion identification. The FACS [1] is a comprehensible taxonomy that provides an objective framework for describing and categorizing the anatomical movements of the face. This coding system comprises 32 non-overlapping fundamental actions, AUs, which correspond to specific individual muscles or groups of muscles. By the same token, the FACS introduces facial AU intensity, which refers to the level or strength of activation of a particular AU. This intensity is important for understanding the magnitude or power of a facial expression. By categorizing AUs into different intensity levels, ranging from slight to extreme, the FACS captures even subtle nuances and variations in facial expressions, which allows for a more precise analysis of emotional states. The measurement and coding of AU intensity follow a scale from 0 to 5. A rating of 0 signifies the absence of any movement, while a rating of 5 represents the maximum intensity or full activation of the specific action unit. The intermediate intensity levels, namely 1, 2, 3, and 4, allow for a detailed representation of facial expressions by capturing varying degrees of muscle activation or movement. Quantifying human facial expressions through AUs allows us to objectively learn, interpret and analyze human emotions in a manner that is both robust and reproducible.

However, the utilization of AUs in research poses challenges as they are usually annotated manually, resulting in time-consuming, repetitive, and error-prone processes. Aiming to address these issues, researchers have focused on building automated ways to detect facial AUs, and research in this direction has gained significant attention. Advancements in automated AU detection have the potential to significantly decrease the time required for this task and enhance the reliability of annotations for various downstream applications. This is closer than ever to being perfected thanks to the continuous advancements in Machine Learning (ML) and Deep Learning (DL) techniques which have emerged as the predominant approaches for handling unstructured data, resulting in remarkable progress in various fields such as computer vision [2], speech recognition [3], and natural language processing [4].

That being said, a significant gap remains in applying ML and DL techniques, such as advanced computer vision ones, to analyze medical data, in particular, when dealing with patients’ information. The inherent nature of the available data, which are often sensitive and confidential, impedes progress in medical research. In practice, medical datasets often contain sensitive biometric information such as facial features, which limits data sharing due to privacy concerns. Healthcare institutions and hospitals, driven by a commitment to preserving patient privacy, are understandably unwilling to share such data, thereby limiting the potential for broader collaborative advancements in the field. This is particularly problematic when analyzing patient facial expressions is required for tasks such as pain assessment, neurodegenerative disease detection, etc. Current studies often rely on original facial images to extract all sorts of relevant information, but the need for non-privacy-invasive methods is increasingly important.

To address these gaps, we propose a lightweight framework for extracting and sharing 3D face landmarks from video recordings and images, which can be used by researchers to strip datasets of confidential content while still enabling AU-based analysis. In addition, we introduce a method for detecting AUs from 3D face landmarks, maintaining high accuracy while avoiding privacy risks. To validate the effectiveness of our approach in a medical setting, we conducted experiments on pain detection. We trained Transformer [5] and Long Short-Term Memory (LSTM) [6] models on AUs extracted from 3D face landmarks in the BP4D+ dataset [7], and achieved results comparable to those obtained when training the models on AUs extracted from the original images (94.9% accuracy vs. 91% accuracy).

This article is a revised and expanded version of a paper titled “Facial Action Unit Detection using 3D Face Landmarks for Pain Detection” [8], which was presented at the IEEE EMBC conference, Sydney, in 2023.

In summary, the contributions of this work can be summarized as follows:

1. We propose a method for AU extraction and intensity estimation from 3D face landmarks.
2. We apply these extracted AUs to the pain detection task, demonstrating their effectiveness in medical applications.
3. We show that a minimal subset of AUs (i.e., 8 out of 34 ground-truth AUs) extracted from 3D face landmarks can be effectively used for pain detection.
4. Overall, we provide a privacy-preserving approach to facial expression analysis, enabling medical researchers to work with facial data while minimizing privacy concerns.

The remainder of this paper is structured as follows: In Section 2, we introduce some important concepts for a better understanding of the current work. In Section 3, we describe some of the work conducted by the research community on the topic of AU detection and facial expression analysis, and introduce our motivation for conducting this research. In Section 4, we describe in detail the proposed method for AU extraction and AU intensity estimation from 3D face mesh as well as the proposed methods for pain detection and dementia detection from AUs. In Section 5, we evaluate the performance of our proposed method and compare it to the conventional ones. Finally, in Section 6, we conclude this paper, and open directions for future work.

## 2. Key Concepts

### 2.1. Action Units

As described in Section 1, AUs are the fundamental components of facial expressions, used in the FACS developed by Ekman and Friesen [1]. Each action unit corresponds to a specific movement of facial muscles, such as raising the eyebrows or pulling the lip corners. AUs are designed to objectively describe facial movements regardless of their emotional interpretation.

The utility of action units lies in their ability to decompose complex facial expressions into smaller, measurable components. This allows for the detailed analysis of human emotions, non-verbal communication, and behavioral patterns. AUs are widely used in psychological research, human–computer interaction, emotion recognition systems, and affective computing. By quantifying facial expressions through AUs, researchers can create datasets for ML, study the dynamics of facial expressions in social contexts, or improve algorithms for recognizing emotional states in real-time applications. Their objectivity and granularity make action units valuable for scientific research, as they enable consistent, replicable studies of facial behavior across diverse fields.

Each AU is assigned a unique identifier and can be associated with multiple emotions. For instance, AU number 9, which represents a nose wrinkle, can indicate emotions such as disgust or pain. In Table 1, we show a compilation of different emotions and their corresponding AUs [9].

### 2.2. Face Mesh

**Figure 1:**
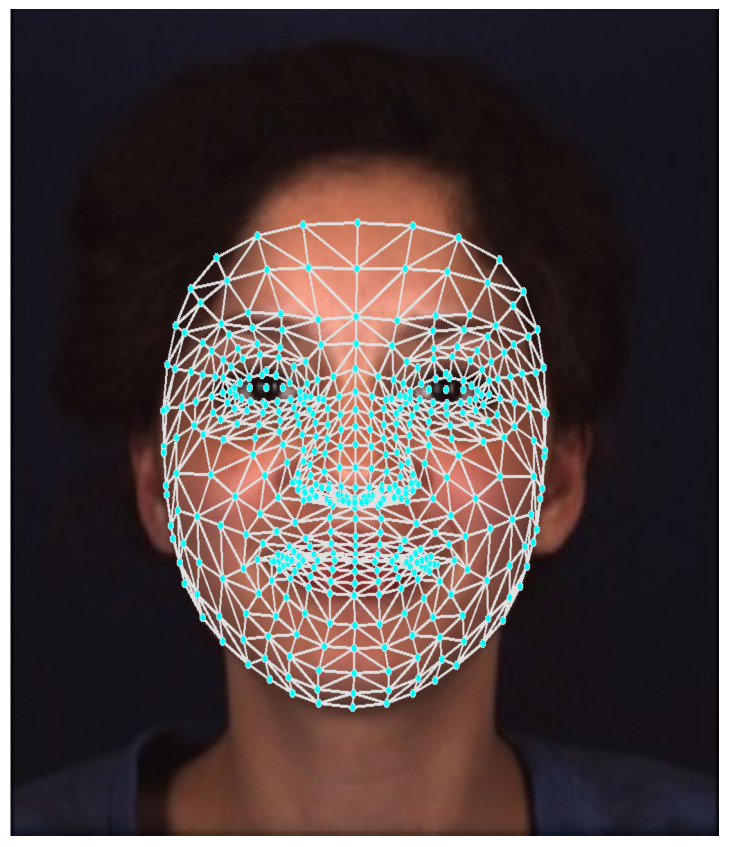
An example of the human face mesh superimposed on the human face itself. Areas around the eyes, the nose, and the mouth have higher landmark density than the remaining parts of the face.

The face mesh is a 3D model of the human face. This type of model is commonly used in applications involving 3D modeling or augmented reality [10]. In our current work, we will be using a pre-trained Neural Network (NN) to extract the face mesh [11]. This NN has been trained to identify the *x* and *y* coordinates of the different landmarks as well as to estimate the *z* coordinate. The model is designed to predict the positions of 468 landmarks spread out across the facial surface, with an additional 10 landmarks allocated for the iris. Overall, the face mesh consists of 478 points, as illustrated in Figure 1.

By converting a face image into a face mesh, we significantly reduce the size of the data by transforming image features into mesh features. This approach retains most of the important and valuable information, allowing for effective facial expression recognition and expression analysis, as we will demonstrate later. Additionally, converting a face image into a face mesh can help preserve the privacy of the individuals participating in any study in which their faces are visible.

### 2.3. Data Augmentation

Different from the two previous concepts, data augmentation is a general technique used in ML and DL to artificially increase the size and diversity of a dataset by applying various transformations such as rotations, flips, or variations in brightness. The purpose of such techniques is usually balancing datasets containing classes of objects with few samples compared to others. In the context of automatic AU detection, data augmentation helps address the issue of rare action units by artificially creating more samples of these underrepresented expressions. By applying the aforementioned transformations to existing data, we can generate synthetic examples of rare AUs, increasing their frequency in the training set.

### 2.4. Pain Detection

Pain is a complicated and subjective experience that profoundly impacts the overall well-being of humans. The development of automatic pain detection systems has the potential to improve quality of life and alleviate suffering for individuals experiencing pain. This advancement has significant implications across a variety of healthcare fields, including medical diagnosis, remote monitoring, and sport medicine.

Being a typical task related to emotion recognition, pain detection is used in this paper to highlight the effectiveness of our methods for face mesh extraction and AU detection.

## 3. Related Work and Motivation

### 3.1. Related Work

#### 3.1.1. AU Detection

Traditional methods for AU detection rely heavily on manually engineered features. For example, Valstar et al. [12] used a Support Vector Machine (SVM) to recognize AUs and analyze their temporal behavior from face videos, utilizing a set of spatio-temporal features calculated from 20 facial fiducial points. Simon et al. [13] introduced kSeg-SVM, a segment-based approach for automatic facial AU detection that combined the strengths of static and temporal modeling while addressing their limitations. By formulating AU detection as a temporal event detection problem, the method modeled feature dependencies and action unit lengths without assuming specific event structures. Experimental results showed that kSeg-SVM outperformed state-of-the-art static methods.

Jiang et al. [14] investigated the use of local binary pattern descriptors for FACS AU detection, comparing static descriptors like local binary patterns (LBPs) and Local Phase Quantization (LPQ). They introduced LPQ-TOP, a dynamic texture descriptor capturing facial expression dynamics, and demonstrated its superiority over LBP-TOP and static methods. Results showed that LPQ-based systems achieved higher accuracy, with LPQ-TOP outperforming all other methods on both posed and spontaneous expression datasets.

Chu et al. [15] addressed the challenge of automatic facial AU detection by proposing the Selective Transfer Machine (STM), a transductive learning method to personalize generic classifiers without requiring additional labels for test subjects. STM reduced person-specific biases by re-weighting relevant training samples while learning the classifier. Evaluations on the Extended Cohn–Kanade (CK+) [16], GEMEP-FERA [17], and RU-FACS [18] datasets showed that STM outperformed generic classifiers and cross-domain learning methods.

Baltrušaitis et al. [19] present a real-time facial AU intensity estimation and occurrence detection framework based on the combination of appearance features (Histogram of Oriented Gradients) and geometry features (shape parameters and landmark locations).

Zhao et al. [20] proposed Deep Region and Multi-label Learning (DRML), a unified deep network for facial AU detection that simultaneously addresses region learning (RL) and multi-label learning (ML). The network features a novel region layer that captures facial structural information by inducing important regions through feedforward functions. DRML’s end-to-end design integrates RL and ML for robust feature learning, achieving state-of-the-art performance on BP4D [21] and DISFA [22] benchmarks in terms of F1-score and AUC.

Recent advancements in DL have yielded impressive results in detecting AUs. Li et al. [23] proposed a DL framework for AU detection that combines Region of Interest (RoI) adaptation, multi-label learning, and LSTM-based temporal fusion. Their method uses RoI cropping nets to independently learn specific face regions, integrates outputs via multi-label learning to capture AU inter-relationships, and optimally selects LSTM layers to enhance temporal feature fusion. Evaluations on the BP4D [21] and DISFA [22] datasets demonstrated significant improvements over state-of-the-art methods, with average performance gains of 13% and 25%, respectively. In [24], they utilized a Gated Graph Neural Network (GGNN) in a multi-scale convolutional neural network (CNN) framework to integrate semantic relationships among AUs.

Shao et al. [25] proposed an end-to-end attention and relation learning framework for facial AU detection that adaptively learns AU-specific features using only AU labels. Their method combines channel-wise and spatial attentions to extract local AU-related features and refines these features through pixel-level AU relations. The framework demonstrated state-of-the-art performance on benchmarks like BP4D [21], DISFA [22], and FERA 2015 [26] for AU detection, AU intensity estimation, and robustness to occlusions and large poses.

In [27], Zhang et al. proposed a model called Multi-Head Fused Transformer that uses both RGB and depth images to learn discriminative AU feature representations. In their work, Jacob et al. [28] utilized image features and attention maps to feed different action unit branches, where discriminative feature embeddings were extracted using a novel loss function. Next, to capture the complex relationships between the different AUs, a Transformer encoder is employed.

#### 3.1.2. AUs for Facial Expression Analysis

The FACS has been widely used for facial expression analysis in various applications. Gupta [29] explored facial emotion detection in both static images and real-time video using the CK and CK+ datasets [16]. Faces were detected with OpenCV’s HAAR filters, followed by facial landmark processing and classification of eight universal emotions using SVM, achieving an accuracy of 93.7%. This approach has applications in human–robot interaction and sentiment analysis, with potential for further improvement in accuracy through landmark refinement.

Darzi et al. [30] investigated the potential of facial action units and head dynamics as biomarkers for assessing Obsessive Compulsive Disorder (OCD) severity, depression, and Deep Brain Stimulation (DBS) energy in patients undergoing DBS treatment for severe OCD. Using Automatic Facial Affect Recognition (AFAR) and clinical assessments, they analyzed data from five patients recorded during interviews across pre- and post-surgery intervals. Their model predicted 61% of OCD severity variation (YBOCS-II [31]), 59% of depression severity (BDI), and 37% of DBS energy variation, highlighting the promise of automated facial analysis in neuromodulation therapy.

Other studies have explored the use of AUs for detecting stress [32,33,34] and pain [35]. Giannakakis et al. [32] proposed a DL pipeline for automated recognition and analysis of facial AUs to differentiate between neutral and stress states. The model uses geometric and appearance facial features extracted from two publicly available annotated datasets, UNBC [36] and BOSPHORUS [37], and applies these to regress AU intensities and classify AUs. Tested on their stress dataset SRD’15, the method demonstrated strong performance in both AU detection and stress recognition. The study also identified specific AUs whose intensities significantly increase during stress, highlighting more expressive facial characteristics compared to neutral states.

Gupta et al. [33] proposed using a multilayer feedforward neural network (FFNN) to distinguish between stress and neutrality based on 17 facial AUs extracted from videos using the Openface tool. The model achieved a prediction accuracy of 93.2% using the CK+ [16] and DISFA [22] datasets, outperforming existing feature-based approaches in distinguishing between the two emotional states.

Bevilacqua et al. [34] proposed a method for analyzing facial cues to detect stress and boredom in players during game interactions. Their approach uses computer vision to extract seven facial features that capture facial muscle activity, relying on Euclidean distances between facial landmarks rather than predefined expressions or model training. Evaluated in a gaming context, their method showed statistically significant differences in facial feature values during boring and stressful gameplay periods, indicating its effectiveness for real-time, unobtrusive emotion detection. This approach is user-tailored and well suited for game environments.

Hinduja et al. [35] trained a Random Forest (RF) model to recognize pain by combining AUs with physiological data. In [38], Meawad et al. proposed an approach for detecting pain in sequences of spontaneous facial expressions, based on extracted landmarks from a mobile device. For comprehensive surveys on automatic pain detection from facial expressions, the readers may refer to [39,40].

### 3.2. Motivation

Given the works described above, the research community has been paying great attention to creating effective tools for detecting AUs which allow for a wide range of benefits in fields such as medicine, psychology, and affective computing.

What sets our proposal apart from conventional methods is the input data format and the simplicity of the NN architecture used for AU extraction and AU intensity estimation. Unlike DL models that process entire images, we only used the 3D face landmarks from the face mesh. Our approach employs a straightforward Fully Connected Neural Network (FCNN) to map 478 facial landmarks to the top 8 AUs. In this sense, our method is more efficient both in terms of computational complexity and execution time.

Nonetheless, throughout this work, we introduce a pipeline that allows researchers to share viable datasets in low-size format and without posing any threat to the privacy of participants. We demonstrate how such datasets can be used effectively for AU extraction, AU intensity estimation, pain detection, and even dementia detection.

The key novel aspects of our works are as follows:

1. Privacy-Preserving AU Extraction Using 3D Face Landmarks: We propose a novel method for extracting facial action units (AUs) and their intensities from 3D face landmarks rather than using original facial images. This not only reduces computational costs but also significantly enhances privacy protection, addressing a critical need in medical research where patient confidentiality is of the utmost importance.
2. Application in Medical Contexts: Our framework is validated through its application to the pain detection task. This typical application demonstrates how the proposed method can be integrated for similar classification tasks. The reported results show that the performance of the proposed method can reach competitive results in computer vision classification tasks, without relying on the full images, but rather on a much smaller distilled information extracted from these images.
3. Competitive Performance with Minimal AU Subset: We show that a minimal subset of AUs (i.e., 8 out of 34 ground-truth AUs) extracted from the 3D face landmarks is sufficient for effective pain detection.
4. Integration of 3D Face Mesh Data: Our method integrates 3D face mesh data, which provide a rich representation of facial structure, enabling more robust AU extraction and intensity estimation. This integration is particularly innovative because it makes use of 3D geometric information to achieve privacy preservation without sacrificing performance.

## 4. Proposed Approach

Before proceeding with our proposed approach for AU extraction, AU intensity estimation, and pain and dementia detection, we will introduce the dataset on which we conducted our experiments.

### 4.1. Dataset

The multimodal BP4D+ dataset [7] offers a rich and diverse collection of modalities, including 3D facial models, RGB images, thermal images, and eight physiological signals (diastolic blood pressure, mean blood pressure, electrical conductivity of the skin, systolic blood pressure, raw blood pressure, pulse rate, respiration rate, and respiration volts). The dataset consisted of 140 participants (82 females and 58 males) engaging in 10 activities aimed at eliciting 10 different emotions. Table 2 shows the details of the activities performed. As shown in [7], when engaging in these activities, the participants have expressed a wide variety of emotions that are in line with the emotions that these activities are meant to elicit. Pain and sadness, in particular, have been consistently reported accurately by all participants when engaging in their respective tasks. Additionally, experts in FACS annotated AUs for four distinct emotion elicitation tasks: happiness, embarrassment, fear, and pain. However, it is important to note that only the most expressive segments were subjected to annotation, resulting in an average duration of approximately 20 s for each segment. We have formulated a binary classification task for pain detection by treating pain sequences as the positive class and the remaining three emotion sequences as the negative class.

In Table 3, we show the distribution of data samples in our subset of BP4D+ [7] that we use in this work. In total, 197,782 frames are used, approximately a third of which are for the pain class. All 140 participants’ data are used for both classes. The data are made of segments of a duration approximately equal to 20 s each.

### 4.2. Overall System Description

**Figure 2:**
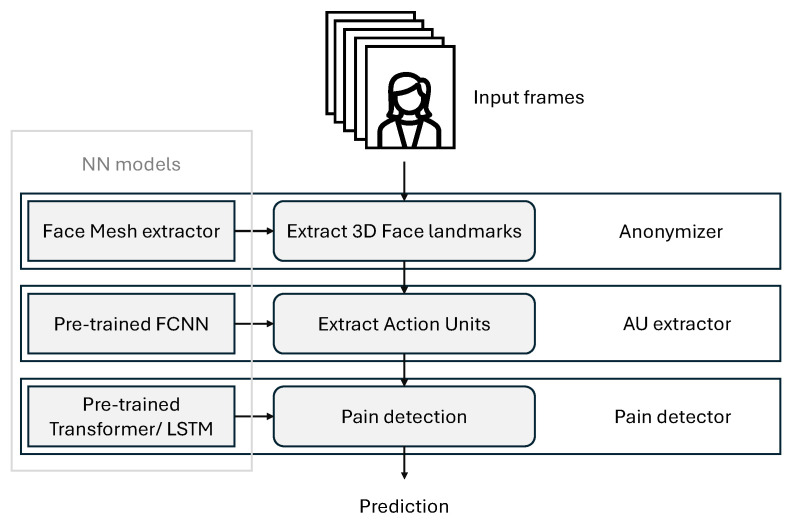
A flowchart of the proposed framework. The framework is composed of 3 main components: an anonymizer, an AU detector, and a pain detector.

**Figure 3:**
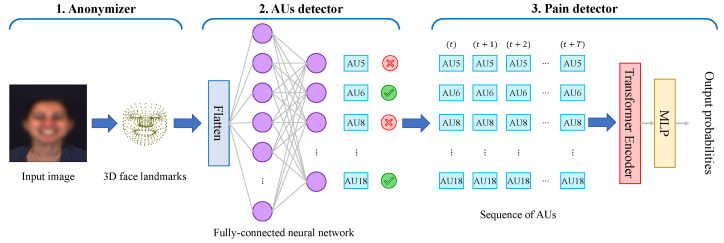
A diagram block of the proposed framework: Upon generating the 3D face landmarks, a 2-layer FCNN with multiple outputs is used to detect the AUs. The sequence of detected AUs is then processed through a Transformer encoder to identify the class (pain).

**Figure 4:**
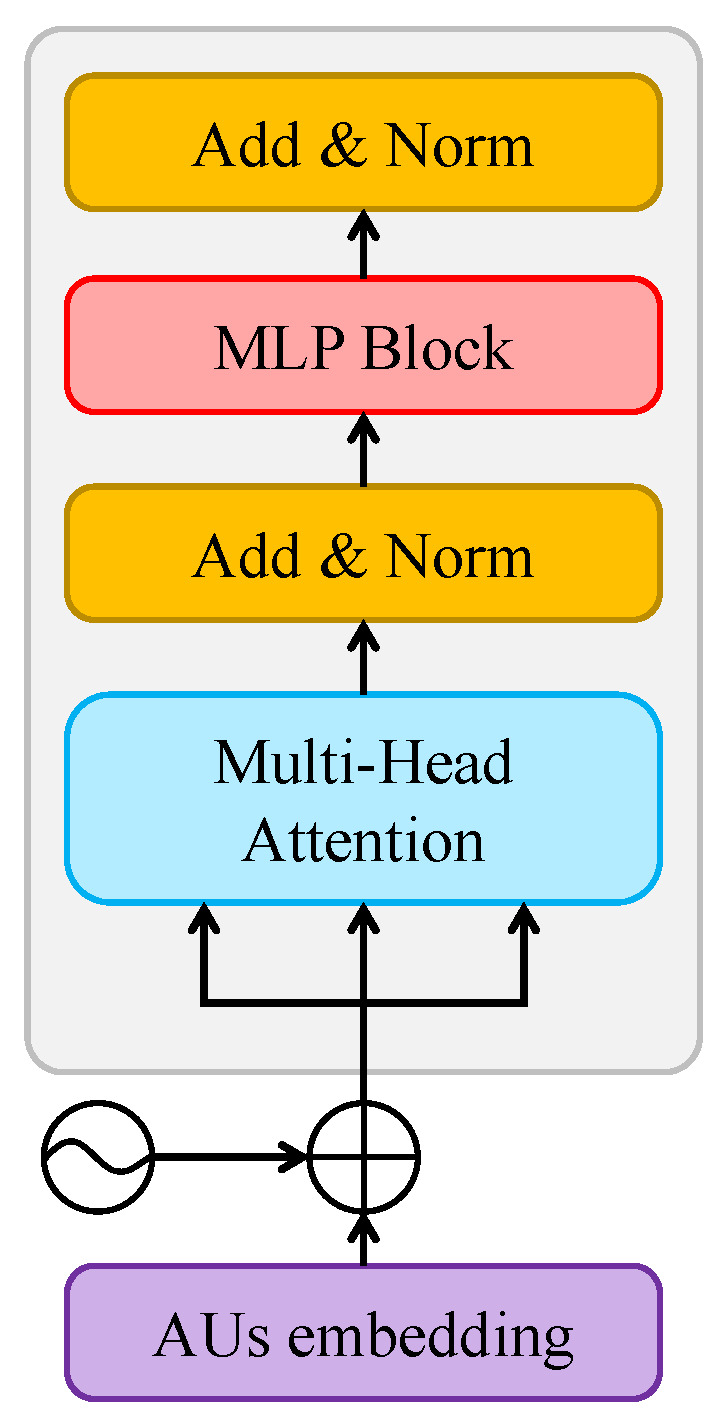
The structure of the Transformer encoder used in our work.

**Figure 5:**
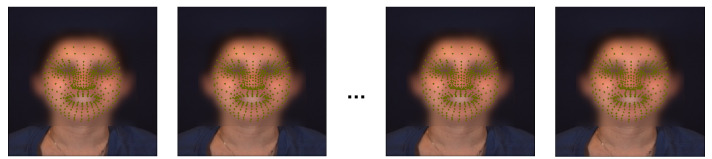
Example of consecutive frames from the dataset (a few seconds apart) along with their detected face landmarks.

In Figure 2, we show the flowchart of our proposed system. In Figure 3, we show a block diagram of the different components of our framework. Further details of the structure of the Transformer encoder block are given in Figure 4. Our system is composed of three main parts:

1. An anonymizer: This component refers to the transformation of images in our dataset into 3D face landmarks. In brief, it generates a set of 478 landmark coordinates over time. Such point coordinates are very useful in extracting information allowing for pain detection, as we will demonstrate, yet they do not allow the identity of the subjects to be identified. In Figure 5, we show a sequence of frames which are a few seconds apart from the dataset along with their detected face landmarks. We employ a pre-trained model [11] for this part. The details of the model and its performance can be found in [11]. In the current paper, we briefly describe how the 3D face landmarks are extracted, how they are formatted, and the performance to expect from this model.
2. An AU detector: This component simply allows the main AUs to be extracted from a given set of 3D face landmarks. Conventionally, NNs are trained to identify AUs from full images, making use of all embedded information. By creating NNs that extract the AUs from the 3D face landmarks, we demonstrate the usefulness of the first component, and that it is still possible to derive relevant information from a condensed and small-size vector such as the set of 3D landmarks.
3. A pain detector: This component relies on the detected AUs to identify the pain expressed by the subjects. It takes the sequence of detected AUs as input and processes them through a Transformer encoder and a Multi-Layer Perceptron (MLP) to identify whether the subject is experiencing pain or not. Although there is still room for improvement in AU estimation accuracy, we show that it is still possible to identify pain with an accuracy comparable to that when using the ground-truth AUs. We also demonstrate that for the pain detection task, very few AUs are required to achieve an accuracy relatively close to that when using all the AUs.

The mathematical formulation of these steps is detailed in Algorithm 1 and in the remainder of this subsection.
**Algorithm 1:** Pain detection using 3D face landmarks.**Require:** RGB image sequence $\left\{I_{1},I_{2},\cdots ,I_{T}\right\}$**Ensure:** Pain expression label y∈{0,1}1:Initialize AU sequence $A_{1:T}\leftarrow \emptyset$2:**for** t=1 to *T* **do**3:   Extract 3D face landmarks: $L_{t}=f_{\theta }\left(I_{t}\right)$4:   Extract AUs from landmarks: $A_{t}=g_{\phi }\left(L_{t}\right)$5:   Append $A_{t}$ to $A_{1:T}$6:**end for**7:Process AU sequence through Transformer Encoder: $Z=\mathrm{TransformerEncoder}(A_{1:T})$8:Predict pain probability: $\hat{y}=\mathrm{MLP}\left(Z_{\mathrm{last}}\right)$9:Return $y=\mathrm{round}(\hat{y})$

### 4.3. Detailed System Description

#### 4.3.1. The Anonymizer

As stated above, this step consists of extracting 3D face landmarks from face images. In our work, we employ the method proposed by Kartynnik et al. [11], which estimates a set of 478 3D landmarks from a single RGB image. This method uses a lightweight convolutional neural network (CNN) with an encoder–decoder architecture.

The encoder consists of a series of depthwise separable convolutional layers, following the MobileNetV2 architecture [41], to efficiently extract spatial features from the input image while reducing computational complexity. It begins with an initial 3×3 convolution, followed by a sequence of depthwise convolutions (which filter spatial information) and pointwise convolutions (which expand feature dimensions), progressively increasing the number of feature maps from 32 to 512 while reducing the spatial resolution. The final bottleneck layer encodes high-level geometric features before passing them to the decoder.

The decoder reconstructs the 3D facial landmarks from these encoded features using transposed convolutional layers, which progressively upsample the feature maps. Starting with 256 filters, the decoder gradually reduces the number of filters while increasing spatial resolution, ultimately producing a structured set of 478 3D landmark coordinates through a fully connected output layer. This architecture ensures an efficient and accurate extraction of facial landmarks, even under variations in pose, lighting, and occlusions.

The model is trained on a large dataset of 3D face scans, with data augmentation techniques ensuring robustness to occlusions, facial expressions, and lighting variations. The output landmarks capture fine-grained facial geometry while removing identifiable features, ensuring privacy preservation. The model achieves real-time performance, running at over 100 FPS on high-end GPUs and 10–30 FPS on mid-range mobile devices. The model reaches a Mean Absolute Distance (MAD) between the predictions and the ground-truth vertex locations, normalized by Interocular Distance (IOD), equal to 2.56%. For further details on the network architecture, training process, and evaluation, we refer the reader to [41].

Mathematically, the input to the anonymizer is an RGB image $I\in R^{H\times W\times 3}$, where *H* and *W* denote the height and width of the image. The pre-trained CNN model $f_{\theta }\left(\cdot \right)$ extracts a set of $N_{s}$ three-dimensional facial landmarks:

(1) $$L=f_{\theta }\left(I\right),\;L\in R^{N_{s}\times 3},$$

where L represents the detected 3D landmarks, and each row $L_{i}=\left(x_{i},y_{i},z_{i}\right)$ corresponds to the 3D coordinates of a landmark.

#### 4.3.2. The AU Detector

Conventionally, AUs are extracted from the images of human faces (similar to the input image to our anonymizer shown in the left part of Figure 3). However, the transformations we applied that allowed us to remove the identity-related information of the participants led to a transformation of the input format itself. As such, rather than using a typical 2D CNN for image processing, in the current work, we use an NN that follows the structure of a typical FCNN. In Figure 3, we show the structure of the proposed network. The network is composed of two dense layers with 128 neurons and 8 neurons, respectively. The input layer’s shape follows the size of the input vector generated for the face landmarks, i.e., a shape of $3\times N_{s}$, with $N_{s}$ referring to the number of landmarks (478 in our case). The output layer composed of 8 neurons is meant to extract 8 AUs (the most common and useful ones for pain detection). It is possible to include more AUs (increase the number of neurons in the output layer). However, other AUs are quite rare in the dataset, and our experiments have shown that it is possible to reach good performance in pain detection using only 8 AUs. The results of the detection will be shown later in Section 5.

Mathematically, given the extracted 3D landmarks L, the AU detector maps them into a set of $N_{a}$ AU intensities using a Fully Connected Neural Network:

(2) $$A=g_{\phi }\left(L\right),\;A\in R^{N_{a}},$$

where $g_{\phi }\left(\cdot \right)$ is a neural network parameterized by weights ϕ. The AU detection network consists of the following:

- Input layer of size $3\times N_{s}$ (corresponding to the 3D landmark coordinates).
- Hidden layer with 128 neurons and ReLU activation.
- Output layer with $N_{a}=8$ neurons, each corresponding to an AU intensity.

#### 4.3.3. The Pain Detector

In this study, we propose using more advanced DL models to detect pain by utilizing the AUs extracted from our AU detector approach as well as all ground-truth AUs. We believe that a customized NN will enhance performance and enable real-time operation. DL is particularly well suited for processing sequential data and eliminates the need for feature engineering, which is often required in traditional ML algorithms. We employed both Transformer and LSTM and compared their performance.

LSTM is a type of Recurrent Neural Network (RNN) that handles sequential data by storing and retrieving information over time. It uses a series of gates to selectively update and forget information at each time step, allowing it to capture long-term dependencies in the input sequence. On the other hand, the Transformer is a type of NN model that has become the go-to method for handling natural language processing tasks. Unlike LSTMs, it does not use recurrent connections, but instead uses self-attention mechanisms to model the relationships between different positions in the input sequence. This allows it to capture dependencies between distant positions in the sequence, which can be difficult for LSTMs. In this work, we utilize only the encoder component of the Transformer model, as shown in Figure 4. The encoder processes AU embeddings, which are combined with a positional encoding and passed through a multi-head attention. This is followed by two blocks of typical Add and Norm and Multi-Layer Perceptron (MLP) which outputs the probabilities for two classes: pain expression and non-pain expression.

Mathematically, in this task, we process a temporal sequence of AU vectors:

(3) $$A_{1:T}=\left\{A_{1},A_{2},\cdots ,A_{T}\right\},\;A_{t}\in R^{N_{a}},$$

where *T* is the number of frames in the sequence. The Transformer encoder processes this sequence as follows:

(4) $$Z=\mathrm{TransformerEncoder}\left(A_{1:T}\right)\in R^{T\times d},$$

where *d* is the embedding dimension. Finally, a Multi-Layer Perceptron (MLP) predicts the probability of pain expression:

(5) $$\hat{y}=\mathrm{MLP}\left(Z_{\mathrm{last}}\right),\;\hat{y}\in \left[0,1\right],$$

where $\hat{y}$ is the probability of pain, and $Z_{\mathrm{last}}$ represents the output of the final frame in the sequence.

## 5. Results and Discussion

### 5.1. Implementation Details

To ensure transparency and facilitate reproducibility, we first provide details about the computational setup used for our experiments. Table 4 summarizes the hardware and software specifications.

Our experiments were conducted on a machine running Windows 11, equipped with an Intel Core i9-11900K processor featuring 16 cores clocked at 3.5 GHz, 64 GB of RAM, and an NVIDIA GeForce RTX 3080 GPU with 10 GB of VRAM. The software environment included Python 3.10.3, TensorFlow 2.9.1, and Keras 2.9.0. This setup provided the computational resources necessary for efficient training and inference of Deep Learning models. For researchers working in resource-constrained environments, alternative setups with lower specifications may still be viable, particularly for inference tasks, though training may require extended time or reduced batch sizes.

#### 5.1.1. AU Detection

A total of 197,782 frames were used for the training and testing. More precisely, we used approximately 66% of the frames for training and the remaining ones for testing. In the context of image processing (such as in the case of the conventional methods), the word ’frame’ refers to a single image extracted from the videos. In the context of our proposed approach, the word ’frame’ refers to the image’s corresponding face mesh. As previously described, the FCNN model is composed of a hidden layer with 128 neurons and a dense layer with 8 neurons for output. As for hyperparameters, we fixed the batch size to 64, the maximum number of epochs to 500, and the learning rate to 0.01.

To validate our AU detection approach, we employed a subject-independent 3-fold cross-validation strategy. Each fold consisted of extracted 3D face landmarks from distinct subjects, which ensured that the model was trained on one set of subjects and evaluated on another set of subjects to ensure generalizability. In terms of performance metrics, we utilized the F1-score to account for imbalanced class distribution.

#### 5.1.2. AU Intensity Estimation

Similar to the AU detection, the AU intensity estimation network is made of a hidden layer with 128 neurons and 5 neurons as output which indicate the intensity of the most present five AUs (i.e., AUs 6, 10, 12, 14 and 17). Note that the number of AUs with high presence in the case of binary labels (i.e., present vs. absent) is not the same as that of AUs with intensity labeling. We used the same batch size and trained the network for the same number of epochs (i.e., 64 and 500, respectively). The learning rate was also set to 0.01.

Similar to AU detection, the AU intensity estimation was performed through a 3-fold cross-validation. The same split was carried out to make sure that subjects used in the evaluation in each fold were different from those in the training. In terms of performance metrics, the Root Mean Square Error (RMSE) and Mean Absolute Error (MAE) between the estimated intensity and the ground-truth one were used.

#### 5.1.3. Pain Detection

Pain detection is treated as a classification task. As such, we used an attention followed by an MLP model as well as an LSTM model for the classification.

During the training process, we used a fixed timestamp of 350 frames, which corresponds to approximately 14 s of data. To determine the optimal parameters for our models, we employed a grid-search strategy. For the Transformer model, we set the dimension of the linear projection layer to 1024, the number of multi-head attention modules to four, the number of encoder layers to two, and the learning rate to $10^{-5}$. Regarding the LSTM model, which is composed of two layers, we fixed the hidden dimension to 512 and the learning rate to $10^{-3}$. Both models were trained with a batch size of 16 and a maximum number of epochs of 150.

All models (for AU and pain detection) were trained using the Adam optimizer [42], with an exponential decay rate for the first- and second-moment estimates fixed at 0.9 and 0.999, respectively. The whole pipeline was implemented using the PyTorch framework [43]. Following prior works [35], we utilized a subject-independent 10-fold cross-validation strategy, where each fold comprised distinct subjects whose AUs were extracted using the AU detection model. For evaluation, we used the accuracy and F1-score.

### 5.2. Results

#### 5.2.1. Action Unit Detection

**Figure 6:**
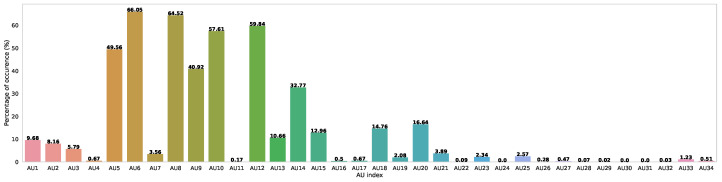
Distribution in percent of the different AUs in our dataset.

As mentioned earlier, numerous AUs were poorly presented in the vast majority of frames. In Figure 6, we illustrate the distribution of AUs within our dataset. As can be seen, a large number of the AUs are almost totally absent in our dataset with a presence ranging from 0.0% (AUs 30 and 31) to a little over 10.66% for AU 13. Overall, the figure clearly demonstrates the imbalance in terms of AU presence in the dataset. Due to the poor presence of many AUs in the dataset, training the network to learn to detect them is unreasonable. As such, our detection results are limited to a subset of eight AUs that are present with relatively enough instances and contribute the most to the task we will address later, namely pain detection. In Table 5, we list these AUs as well as their FACS name, which indicates which facial muscle is moved. In addition to not having great contribution to pain detection, the infrequent AUs represent only a very small portion of the ground truth; thus, they cannot be used to identify pertinent or generalizable patterns, as we will demonstrate later on.

With that in mind, we present in Table 6 the average F1-score (%) of detection of the top eight present AUs, which also happen to be the most useful ones in our study. The table shows the results of AU identification with our proposed method using 3D face mesh and that using a pre-trained CNN applied to the original images from the dataset. In the table, FCN (BP4D+) refers to applying our fully connected network to the 3D facial landmarks in the BP4D+ dataset, and FCN (Mediapipe) refers to the 3D facial landmarks extracted using Mediapipe [11]. Unlike Mediapipe [11], the 3D facial landmarks in BP4D+ are composed of only 83 points. Since they are annotated by experts, they are more accurate than the ones obtained using a tool like Mediapipe. However, despite that, our proposed method using Mediapipe reaches F1-scores that are superior to the ones obtained when we use the 3D face landmarks from BP4D+. For instance, the detection of AU8 using our proposed method reaches an F1-score equal to 89.7%, outperforming the detection using FCN (BP4D+), which only reaches 84.4%. Other AUs exhibit similar behavior, with our method reaching 86.27% and 75.27% in detecting AUs 6 and 14, outperforming the detection using FCN (BP4D+) by a clear margin, which reaches an F1-score equal to 84.03% and 72.90%, respectively, for these AUs. Overall, for all eight AUs, our method consistently outperforms FCN (BP4D+) with improvement ranging from 0.33% for AU18 to 4.70% for AU8.

In addition to the detection using face landmarks, and for a proper comparison with the detection when using the original RGB image, we also report the results of the AU detection using a pre-trained CNN (ResNet50 [44]) applied to these images in their original format. Applying such a deep NN is obviously pushing the detection ability to its limit, which could be seen as an upper bound for what the detection could reach (using our method). The detection accuracy for our proposed method ranges from 55.13% for AU18 to 89.1% for AU8, with an overall average F1-score equal to 79.25%. This is not far from the detection using ResNet50 [44], whose performance ranges from 59.99% for AU18 to 89.12% for AU10 with an average equal to 82.34%. More interestingly, for AUs 8, 12, and 14, our method shows very competitive detection, outperforming the detection using REsNet50, where the F1-scores reach 89.10%, 80.10%, and 75.27%, respectively.

Another point worth mentioning is the poor detection results for AU18, which reached 55.13% using our method, and 59.99% using ResNet50. The main reason behind the poor detection accuracy of AU18 for all the methods is its low presence in the dataset. As opposed to the other seven AUs identified here, AU18 is present in only 14.76% of the frames in our dataset. That being said, the performance is overall good for our method which relies on a limited number of face landmarks, as opposed to other DL models, which process the whole images. More importantly, as we will demonstrate, the detected AUs from the human face landmarks can be used to perform classification tasks such as pain detection.

In comparison to the extraction of the top eight AUs, the remaining AUs have a much lower presence, rendering their detection quite challenging. Despite that, in our work, we try to detect the next seven more present AUs, namely AUs 1, 2 7, 15, 20, 23, and 25. To address the issue of their low presence, we need a means to increase the number of instances in which they occur. In this context, we employ the technique we previously introduced (i.e., data augmentation) by introducing copies of the original images (from the training subset only) with several alterations including horizontal flip, rotation, cropping and linear distortion. In Table 7, we show the detection of the aforementioned seven AUs with and without augmentation using our proposed method. As can be observed, the overall detection of AUs is much poorer than that of the AUs that are more common in the dataset. That being said, the performance after augmentation shows clear improvement. The overall average AU detection went from 16.31% to 31.16%, with AU20 and AU15 reporting 49.43% and 43.97%, respectively. Again, this is not very far from the results obtained using ResNet50 [44], where the results range from 24.2% to 53.2% for AU23 and AU20, respectively.

Accounting for the augmentation, the detection of AUs 15 and 20 reaches an F1-score equal to 40.13% and 42.20%, respectively, using our proposed method. Using ResNet50 [44], the detection of these AUs reaches an F1-score equal to 46.97% and 53.23%, respectively. While our method lags behind ResNet50 in the detection of these secondary AUs, it is important to recall that ResNet50 is originally trained on a much larger dataset made of over 1.2 M images and has learned very intricate patterns, and therefore can identify very subtle nuances in human facial expressions that are hard to identify using 3D face landmarks.

**Figure 7:**
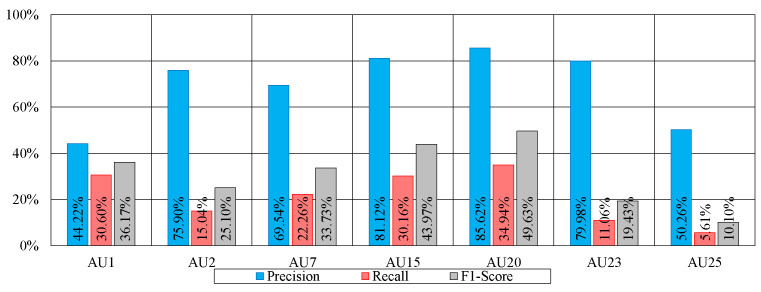
Precision, recall and F1-scores of the detection of the secondary AUs.

To further investigate the performance for each of these AUs, we show in Figure 7 the precision and recall as well as the already reported F1-score of the method FCN (BP4D+). Since both precision and recall contribute to the F1-score, our goal is to highlight which of the two metrics contributes more to the obtained results. As can be seen, for all six AUs, the precision is much higher than the recall. For instance, the precision scores for AUs 2, 15, 20, and 23 reach 75.90%, 81.12%, 85.62%, and 79.98%, respectively. The recall for these same AUs reaches 15.04%, 30.16%, 34.94%, and 11.06%, respectively. The low recall indicates that for these AUs, in many frames, they have not been detected. This is in part due to the low presence of these AUs in the training set, leading the model to learn few patterns that help detect them. In other words, the model did not encounter enough samples in the training set to understand how to detect them correctly. More interesting are the high precision levels reported. A high precision indicates that when detected, an AU is very likely to be indeed present. In other words, even though the model cannot always identify these AUs, when they are detected, the model is highly likely to be correct.

#### 5.2.2. AU Intensity Estimation

**Figure 8:**
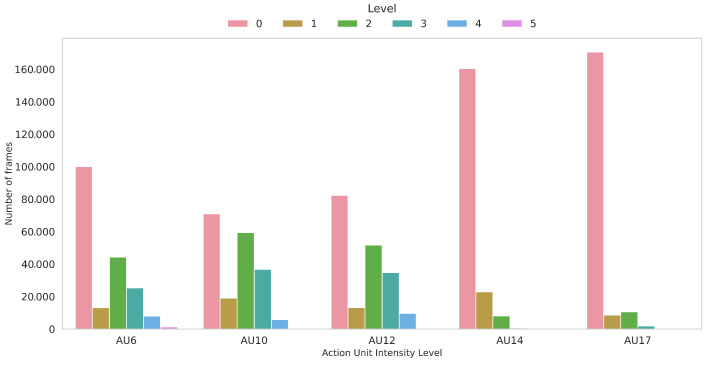
Distribution of the intensity level for each action unit in our dataset.

The AU intensity estimation is an even more challenging task than the AU detection. This is because it does not consist of a binary classification, but rather of identifying one of multiple levels. In addition, subjects with different ages have different manifestations of their facial muscle movements which renders the estimation an even harder task. Nevertheless, the presence of different intensities is even lower than the presence of binary AUs in the dataset. In Figure 8, we show the top five most present AUs with different intensities in our dataset. As can be observed, intensity 0 (which indicates absence) is predominant, with AUs such as AU14 and AU17 appearing in much fewer samples when their intensities are non-equal to 0.

That being said, in our work, we address the AU intensity estimation as a regression task, by which our NN tries to minimize the difference between the predicted intensity and the ground-truth one. This means that, while the AU intensity should take discrete values, our model predicts a continuous value between 0 and 5. For a realistic prediction, it is possible to simply round the estimated value to its nearest integer one. As stated above, we use the same NN we previously introduced for AU estimation with the difference that our output layer consists of five neurons with a linear activation.

In Table 8, we report the results of AU intensity estimation using our proposed method, two baseline ones including a random guess and a majority class, as well as the estimation using ResNet50 [44] fine-tuned for the estimation of individual AUs (one at a time).

As can be seen, our method outperforms the baseline ones by far, even the majority class one, reaching an RMSE equal to 0.66 and an MAE equal to 0.44. Despite the intensity 0 being dominant for all AUs in the dataset, our method outperforms the majority guess with an improvement of 0.67 and 0.41 in RMSE and MAE, respectively. On the other hand, our method lags behind ResNet50 [44] in both RMSE and MAE, with a difference of 0.08 and 0.09 in those metrics, respectively. Overall, the skewness of data in favor of the intensity 0 renders drawing a conclusion about the overall performance and its generalizability challenging. Further investigations are needed to confirm the effectiveness of both our proposed method and ResNet50 [44] and how well they perform on other datasets.

#### 5.2.3. Pain Detection

We present the performance of the pain detection task using four different settings, which are as follows:

- Eight predicted AUs (8AUP), in which the top eight predicted AUs (see Table 5) are used for pain detection
- Eight ground-truth AUs (8AUG), in which the ground truth of the same 8AUP are used for pain detection
- All ground-truth AUs (All-AUG), in which all the 34 ground-truth AUs are used for pain detection
- Using ResNet50 [44] + LSTM, in which the ResNet model is fine-tuned on the actual images from the dataset, and the output probabilities of each image are fed to an LSTM similar to ours.

In Table 9, we show the performance for the task of pain detection. We report the overall accuracy and F1-score for each of the four experiments. As we can see, the results of training Transformer and LSTM models with 8AUP and 8AUG show a marginal difference. The detection F1-score for 8AUP reaches 82.10% using the Transformer model, and 81.50% using the LSTM model. This is not very far from the prediction using 8AUG as the difference in F1-score is only 0.26% and 0.09% in favor of 8AUG, respectively. When comparing 8AUP with All-AUG, the difference is slightly higher. The detection using All-AUG reaches an F1-score equal to 84.53% using the Transformer model and 82.63% using the LSTM model. Despite using fewer AUs in total when employing the method 8AUP, the difference is only 2.43% and 1.13% in terms of F1-score, and 1.30% and 0.58% in terms of accuracy in favor of All-AUG for Transformer and LSTM, respectively.

In addition, 8AUP outperforms the Random Forest (RF) model proposed in [35]. To the best of our knowledge, the work [35] is the only one in the literature which addresses the same task as ours directly, and can be used as a baseline for a direct comparison to our work. Our approach results in a significant improvement in F1-score and accuracy, by 8.7% and 1.79% when using Transformer, and by 8.1% and 2.14% when using LSTM, compared to the Random Forest model. When using All-AUG, the F1-score and accuracy are improved by 11.13% and 2.72% when using Transformer, and by 9.23% and 2.14% when using LSTM, compared to the Random Forest model. As stated above, we also compare our method to ResNet50+LSTM, which allows us to assess how far these AUs can go compared to when using full sequences of images. Table 9 shows that our method is only 2.76% behind accuracy-wise and 2.01% F1-score-wise when using all ground-truth AUs. When using the predictions of our earlier model, the accuracy is 4.06% below that of ResNet50+LSTM, and the F1-score is 6.44% below.

We then explore the confusion matrix of the classification using the method 8AUP. The confusion matrix as shown in Table 10 summarizes the classification performance for the pain detection task using this method. Overall, we can see that we have a well-balanced confusion matrix, indicating that the pain detection model is performing well for both classes (pain vs. no pain), and is not biased towards one class or the other. This aspect is particularly important when working with datasets that exhibit significant class imbalances, as is common in medical datasets where images showing symptoms of a condition are often far fewer than those of healthy subjects.

### 5.3. Discussion

#### 5.3.1. Performance Analysis and Computational Efficiency

Our proposed method demonstrates promising results in AU detection, AU intensity estimation, and downstream tasks such as pain detection. However, while the approach is effective, there are areas for further enhancement. Specifically, detecting AUs and estimating their intensities from 3D facial landmarks are inherently more challenging than using raw images, as the latter captures fine-grained details and subtle facial muscle movements. Consequently, we treat image-based methods, such as ResNet50 [44], as an upper bound for performance comparison.

Despite this limitation, our method achieves results that are remarkably close to this upper bound. For instance, in the detection of the top eight AUs, our approach reaches an F1-score of 79.25%, only 3.09% behind ResNet50. Similarly, for pain detection, our method attains an accuracy of 92.11% and an F1-score of 84.53%, which are only 2.76% and 4.01% lower than those obtained by ResNet50. Importantly, our method operates with significantly fewer computational resources. While ResNet50 comprises 25.6 million parameters, our AU detection model consists of only two layers with 128 and 8 neurons, respectively, resulting in a much smaller network with only a few thousand parameters. This lightweight design makes our approach more efficient and suitable for privacy-preserving applications where raw image sharing is not desirable.

#### 5.3.2. Novelty and Contribution Compared to Existing Work

Compared to prior studies on AU detection and pain estimation, our work introduces a novel approach that prioritizes privacy-preserving facial analysis by relying solely on 3D face landmarks rather than raw images. While conventional DL-based AU detection methods extract features from entire facial images, our approach uses an anonymized representation of facial expressions, ensuring that sensitive identity-related information is not exposed. This design makes our method particularly useful in privacy-sensitive applications, such as medical diagnostics and human–computer interaction, where data security is of the utmost importance.

Another key distinction from previous work is the explicit comparison between AU detection using raw images and using 3D face landmarks. Our results demonstrate that, although raw images provide slightly higher accuracy, our landmark-based approach remains highly competitive. The proposed method also employs a simplified neural network architecture that significantly reduces computational overhead while maintaining reliable performance, making it feasible for deployment in resource-constrained environments.

#### 5.3.3. Implications for Stakeholders and Future Directions

The findings of this study have several implications for both researchers and industry stakeholders. First, the demonstrated ability to extract meaningful AU information from anonymized 3D face meshes is a step towards privacy-aware affective computing applications. This is particularly relevant in fields such as healthcare and remote patient monitoring, where privacy concerns often limit the use of traditional computer vision techniques. Additionally, our work highlights the feasibility of using minimal feature representations for AU-based affect analysis, which could influence future research directions in lightweight and privacy-preserving emotion recognition.

Moving forward, several areas can be explored to enhance the robustness and applicability of our method. One primary limitation is the dependence on the quality of the input images for accurate face mesh extraction. Low-quality images can lead to errors in face landmark estimation, which in turn affect AU detection accuracy. This issue is further exacerbated in video sequences, where consistency across frames is crucial. Future work should include evaluating the method on datasets with more diverse image quality levels to better assess its robustness.

Additionally, while our approach currently relies on a pre-trained face mesh extraction model [11], incorporating a fine-tuned version specifically optimized for AU detection could potentially improve performance. Another direction for improvement is to explore hybrid approaches that integrate both raw image-based and landmark-based methods to leverage the strengths of both representations.

#### 5.3.4. Limitations and Future Research Directions

Despite its advantages, our method has certain limitations. First, the transformation of facial images into 3D landmarks results in some loss of detailed facial information, which can affect AU estimation accuracy. While our results show that this loss is minimal, further improvements can be made by refining the AU detection model using more advanced geometric feature representations. However, it is important to note that for the task at hand (i.e., pain detection) the relevant AUs are typically intense and prominently expressed on the face. This makes their detection from 3D face landmarks relatively easier. In contrast, other tasks may involve more subtle AU activations, making their detection from face landmarks significantly more challenging. On the other hand, detecting such subtle AUs from images remains feasible to some extent, as deep NNs like ResNet50 can capture fine facial variations and associate them with the corresponding AUs.

Second, the approach is currently evaluated on a controlled dataset with high-quality images. A more comprehensive evaluation on real-world, in-the-wild datasets with varying lighting conditions, occlusions, and camera angles would be necessary to determine its robustness across different scenarios.

Lastly, while our current neural network architecture is lightweight and efficient, exploring alternative feature representations or hybrid approaches that integrate both geometric and texture-based information that are also non-privacy-invasive could further enhance AU detection. Additionally, optimizing model training with larger and more diverse datasets may help bridge the performance gap between landmark-based and image-based AU detection methods.

## 6. Conclusions and Future Work

In this paper, we introduced a novel approach for AU detection using only 3D facial landmarks, eliminating the need for raw facial images. Our method achieved an overall F1-score of 79.25% for the top eight AUs in the multimodal BP4D+ dataset [7] and demonstrated competitive performance in AU intensity estimation. By using only the extracted AUs, we further validated our approach on the downstream task of pain detection, where Transformer and LSTM-based models trained on the predicted AUs achieved results comparable to those trained on ground-truth AUs. This highlights the effectiveness of our AU detection model in preserving critical facial expression information.

Compared to existing works, our approach provides a privacy-preserving alternative to image-based AU detection, while maintaining strong predictive performance. Despite a slight drop in accuracy compared to the ResNet50-based model [44] which processes full facial images, our method significantly reduces computational complexity by using only a small set of 3D landmark coordinates. Furthermore, our results demonstrate that even when using only eight predicted AUs, our framework outperforms existing baseline methods for pain detection on the BP4D+ dataset [7].

This work contributes to the broader research community by providing an efficient, lightweight, and privacy-conscious solution for sharing medical datasets without compromising the privacy of the patients while achieving decent performance. Future work will focus on improving the detection of AUs with low presence, refining AU intensity estimation, and developing a more sophisticated multi-task learning framework to enhance both AU detection and intensity estimation simultaneously. These advancements will further close the gap between landmark-based and image-based AU recognition, leading to more practical and privacy-aware facial expression analysis systems.

## Figures and Tables

**Table 1:** Examples of emotions and their associated AUs.

Emotion	AUs
Happiness	6, 7, 12, 25, 26
Sadness	1, 4, 6, 15, 17
Fear	1, 2, 4, 5, 7, 20, 25
Anger	4, 5, 17, 23, 24
Disgust	7, 9, 19, 25, 26
Pain	4, 6, 7, 9, 17, 18, 23, 24

**Table 2:** The ten emotions eliciting tasks that were used during the collection of data to build the BP4D+ dataset.

Task	Activity Performed	Emotion Elicited
T1	Listen to a funny joke	Happiness
T2	Watch a 3D avatar	Surprise
T3	911 phone call	Sadness
T4	Sudden burst of sound	Startle
T5	Interview: true or false question	Skeptical
T6	Silly song	Embarrassment
T7	Physical threat	Fear
T8	Hand into ice water	Pain
T9	Interview: complained about a poor performance	Angry
T10	Smelly odor	Disgust

**Table 3:** The structure of the subset of BP4D+ [7] used in our work.

Class	Pain	Others
Number of participants	140	
Age range	18–66	
Sex ratio (M/F)	58 / 82	
Number of ethnicities	5	
Segment length	∼20 s	∼20 s
Number of segments/participant	1	3
Total number of frames	50,250	147,532

**Table 4:** Hardware and software specifications used for our experiments.

Component	Specification
Operating System	Windows 11
CPU	Intel Core i9-11900K (16 cores, 3.5 GHz)
RAM	64 GB
GPU	NVIDIA GeForce RTX 3080 (10 GB VRAM)
Python Version	3.10.3
Deep Learning Framework	TensorFlow 2.9.1, Keras 2.9.0

**Table 5:** The top 8 present AUs in the dataset and their respective FACS names.

AU Number	FACS Name
AU 5	Upper lid raiser
AU 6	Cheek raiser
AU 8	Lips toward each other
AU 9	Nose wrinkler
AU 10	Upper lip raiser
AU 12	Lip corner puller
AU 14	Dimpler
AU 18	Lip pucker

**Table 6:** The individual and average F1-score (%) of the detected AUs with our proposed method.

Method	AU5	AU6	AU8	AU9	AU10	AU12	AU14	AU18	AVG
FCN (BP4D+)	82.13	84.03	84.40	76.70	86.57	78.30	72.90	54.80	77.85
FCN (Mediapipe)	82.87	86.27	89.10	77.43	87.83	80.10	75.27	55.13	79.25
ResNet50 [44]	85.44	85.22	87.88	79.11	89.12	79.19	74.74	59.99	82.34

**Table 7:** F1-score (%) for 7 AUs using data augmentation with our proposed method.

Method	AU1	AU2	AU7	AU15	AU20	AU23	AU25	AVG
No Augmentation	17.11	18.02	14.21	24.55	31.11	4.98	4.17	16.31
FCN (BP4D+)	36.17	25.10	33.73	43.97	49.63	19.43	10.10	31.16
FCN (Mediapipe)	33.63	27.47	25.53	40.13	42.20	16.73	13.53	28.46
ResNet50 [44]	39.87	31.21	35.84	46.97	53.23	25.20	24.20	36.65

**Table 8:** RMSE and MAE of AU intensity estimation results using our method as well as a baseline one.

Method	Metric	AU6	AU10	AU12	AU14	AU17	AVG
Majority class	RMSE	1.68	1.87	1.93	0.54	0.58	1.32
	MAE	1.08	1.42	1.40	0.21	0.17	0.85
Random guess	RMSE	2.57	2.36	2.43	2.90	2.95	2.64
	MAE	2.07	1.90	1.96	2.36	2.42	2.14
ResNet50 [44]	RMSE	0.57	0.61	0.60	0.64	0.50	0.58
	MAE	0.49	0.51	0.48	0.14	0.12	0.35
Proposed method	RMSE	0.72	0.72	0.75	0.52	0.57	0.66
	MAE	0.61	0.60	0.63	0.20	0.16	0.44

**Table 9:** Pain detection performance using 8AUP as well as the ground-truth ones.

	8AUP			8AUG			All-AUG			Images		
Method	F1	Acc		F1	Acc		F1	Acc		F1	Acc	
RF [35]	-	-		-	-		73.40	89.02		-	-	
LSTM	81.50	91.16		81.59	91.03		82.63	91.74		-	-	
Transformer	82.10	90.81		82.36	90.89		84.53	92.11		-	-	
ResNet50 + LSTM	-	-		-	-		-	-		88.54	94.87	

**Table 10:** Sum of all the confusion matrices for the task of pain detection over the 10 folds using the 8 predicted AUs (8AUP).

Class	Classified as	
	Others	Pain
Others	**387**	22
Pain	29	**117**

## Data Availability

The dataset used in this work can be obtained by contacting the authors of the work [7].

## Abbreviations

The following abbreviations are used in this manuscript:

AFAR	Automatic Facial Affect Recognition
AI	Artificial intelligence
AU	Action unit
CK+	Extended Cohn–Kanade
DBS	Deep Brain Stimulation
DL	Deep Learning
DRML	Deep Region and Multi-label Learning
FACS	Facial Action Coding System
FCNN	Fully Connected Neural Network
FFNN	Feedforward neural network
GGNN	Gated Graph Neural Network
LBP	Local binary pattern
LPQ	Local Phase Quantization
LSTM	Long Short Term Memory
MAE	Mean Absolute Error
ML	Machine Learning
MLL	Multi-Label Learning
MLP	Multi-Layer Perceptron
NN	Neural Network
OCD	Obsessive Compulsive Disorder
RF	Random Forest
RL	Region Learning
RMSE	Root Mean Square Error
RNN	Recurrent Neural Network
ROI	Region of Interest
STM	Selective Transfer Machine
SVM	Support Vector Machine
